# FOXP1 Promotes Spermatogonial Stem Cell Proliferation by Transcriptionally Repressing GSK3B to Activate the Wnt/β‐Catenin Signaling Pathway

**DOI:** 10.1155/sci/1109631

**Published:** 2026-10-19

**Authors:** Jin-Hua Wu, Qing Feng, Feng Lin, Jiang-Ping Xie, Pei-Yan Liu, Yan-Hua Wang, Hui-Liang Zhou

**Affiliations:** ^1^ Department of Andrology and Sexual Medicine The First Affiliated Hospital of Fujian Medical University Fuzhou China fjmu.edu.cn; ^2^ Reproductive Medicine Department Ganzhou Hospital-Nanfang Hospital Southern Medical University (Ganzhou People’s Hospital) Ganzhou China fimmu.com; ^3^ Department of Andrology Ganzhou Hospital-Nanfang Hospital Southern Medical University (Ganzhou People’s Hospital) Ganzhou China fimmu.com

**Keywords:** FOXP1, GSK3B, nonobstructive azoospermia, spermatogonial stem cells, Wnt/β-catenin signaling

## Abstract

**Background:**

Nonobstructive azoospermia (NOA) is characterized by impaired spermatogenesis, yet the molecular mechanisms regulating spermatogonial stem cells (SSCs) in NOA remain unclear. Forkhead box protein P1 (FOXP1), a transcription factor implicated in cell proliferation and differentiation, may play a critical role in SSC function.

**Methods:**

Testicular tissues from OA and NOA patients were examined for FOXP1 expression using reverse transcription‐polymerase chain reaction (RT‐PCR) and immunohistochemistry (IHC). Cell viability, proliferation, and apoptosis were assessed by CCK‐8, EdU, and terminal deoxynucleotidyl transferase dUTP nick end labeling (TUNEL) assays. The interaction between the FOXP1 and GSK3B promoter was analyzed via bioinformatics, chromatin immunoprecipitation, and luciferase reporter assays. Western blotting evaluated Wnt/β‐catenin pathway activation. The role of this pathway was further confirmed by LiCl treatment in FOXP1‐silenced SSCs.

**Results:**

FOXP1 was downregulated in NOA samples displaying defective spermatogenesis. FOXP1 knockdown inhibited SSC viability and proliferation while promoting apoptosis. Mechanistically, FOXP1 bound directly to the GSK3B promoter, repressing its transcription. Silencing FOXP1 increased GSK3B expression and suppressed Wnt/β‐catenin signaling. Activation of Wnt/β‐catenin by LiCl reversed the detrimental effects of FOXP1 knockdown on SSCs.

**Conclusion:**

FOXP1 positively regulates SSC proliferation and survival by transcriptionally repressing GSK3B and maintaining Wnt/β‐catenin signaling. These findings highlight that the FOXP1‐GSK3B‐Wnt/β‐catenin signaling axis plays an essential role in maintaining SSC homeostasis and spermatogenic function and provides novel mechanistic insights into the molecular pathogenesis of NOA.

## 1. Introduction

Nonobstructive azoospermia (NOA) is a major cause of male infertility characterized by a failure of spermatogenesis, leading to the absence of sperm in the ejaculate despite unobstructed reproductive tracts [1, 2]. Unlike obstructive azoospermia (OA), where sperm production is typically normal, NOA results from intrinsic defects within the testicular environment, including disruptions in the regulation and maintenance of spermatogonial stem cells (SSCs) [3, 4], which are essential for continuous sperm generation throughout life. Understanding the molecular mechanisms that govern SSC function in NOA is critical as current treatments remain limited and largely ineffective for many affected individuals.

SSCs represent a rare population within the testicular niche, endowed with the capacity for self‐renewal and differentiation into mature spermatozoa [5, 6]. The delicate balance between SSC proliferation and differentiation is tightly controlled by a complex interplay of intrinsic genetic programs and extrinsic signaling cues [7]. Dysregulation of these regulatory networks can lead to impaired SSC maintenance, apoptosis, or aberrant differentiation, ultimately compromising spermatogenesis and fertility [7]. A variety of transcription factors have been identified as key modulators of SSC biology, orchestrating gene expression patterns that maintain stemness or promote lineage commitment [8]. Among these, forkhead box protein P1 (FOXP1), a member of the FOXP family of transcription factors, has attracted increasing attention due to its established roles in directing cell proliferation, differentiation, and apoptosis in diverse tissues [9].

FOXP1 is evolutionarily conserved and functions primarily as a transcriptional regulator through DNA binding and interaction with other cofactors [10]. It has been implicated in developmental processes and maintenance of stem cell populations in various organs [11], but its precise role in SSC biology and spermatogenesis remains insufficiently explored. Given the centrality of SSCs in sustaining spermatogenesis, investigating how FOXP1 influences SSC function could reveal novel mechanisms underlying NOA pathogenesis and identify potential therapeutic targets. Emerging evidence highlights the importance of the Wnt/β‐catenin signaling pathway in regulating SSC proliferation and differentiation [12]. Activation of this pathway is known to promote SSC self‐renewal and maintain stem cell pools, thereby supporting continuous sperm production [13]. Glycogen synthase kinase 3 beta (GSK3B) acts as a critical negative regulator of Wnt/β‐catenin signaling by promoting β‐catenin degradation. Modulation of GSK3B expression or activity is thus pivotal for controlling downstream Wnt signaling dynamics [14]. However, how transcription factors such as FOXP1 integrate into this signaling axis within SSCs remains poorly understood.

This study aimed to elucidate the role of FOXP1 in SSC biology and its potential regulatory relationship with GSK3B and the Wnt/β‐catenin pathway. We investigated FOXP1 expression in testicular tissues from NOA and OA patients, revealing significantly decreased FOXP1 levels in NOA accompanied by a compromised spermatogenic morphology. Mechanistic analyses identified the direct binding of FOXP1 to the GSK3B promoter, resulting in transcriptional repression of GSK3B. Reduced FOXP1 led to increased GSK3B expression, attenuated Wnt/β‐catenin signaling, and impaired SSC function. Collectively, these findings position FOXP1 as a key transcriptional regulator sustaining SSC proliferation and survival by repressing GSK3B and maintaining Wnt/β‐catenin signaling activity. Deciphering this regulatory axis enriches our understanding of the molecular basis of spermatogenic failure in NOA and highlights FOXP1 as a potential therapeutic target for restoring fertility in affected men.

## 2. Materials and Methods

### 2.1. Clinical Samples and Ethical Approval

Testicular tissue specimens of OA (*N* = 18) and NOA (*N* = 13) were collected from patients who underwent surgical resection at Ganzhou People’s Hospital of Jiangxi Province. All enrolled NOA patients were clinically diagnosed as idiopathic after the exclusion of known genetic causes. The exclusion criteria are as follows: abnormal karyotype (e.g., Klinefelter syndrome); Y‑chromosome AZF microdeletions; history of chemotherapy/radiotherapy; known monogenic infertility‑related mutations from clinical genetic screening; cryptorchidism history. OA patients had normal spermatogenesis confirmed by testicular histology, with obstruction as the only cause of azoospermia. Testicular histopathology of each NOA patient was evaluated and classified according to standard classification: Sertoli‑cell‑only syndrome (SCO), maturation arrest (MA), and hypospermatogenesis (HS). Table S1 represented individual patient information, including patient ID, group (OA/NOA), age, and histopathological subtype. For each patient, two separate biopsy fragments were obtained from different regions of the unilateral testis. One fragment was fixed in paraformaldehyde for HE staining and immunohistochemistry (IHC). The second parallel fragment was snap‑frozen at −80°C for RNA extraction and RT‑PCR analysis. Specimens were maintained in a low‐temperature refrigerator (−80°C) for gene and protein examination. This study was approved by the Ethics Committee of Ganzhou People’s Hospital of Jiangxi Province (Approval Number PJB2025‐279‐01), and all patients provided written informed consent in accordance with the Declaration of Helsinki.

### 2.2. Hematoxylin–Eosin (HE) Staining

Paraformaldehyde‐fixed testicular tissues were dehydrated through a gradient ethanol series, embedded in paraffin, and sectioned into 5‐μm‐thick slices. The sections were deparaffinized with xylene and rehydrated and then stained with hematoxylin (Solarbio, G1120, Beijing, China) for 5 min and eosin (Solarbio, G1121, Beijing, China) for 30 s. After dehydration and transparency, the sections were mounted with neutral balsam (Sigma‐Aldrich, 01381, St. Louis, MO, USA) and observed under an optical microscope (Olympus, BX53, Tokyo, Japan). Images were captured and analyzed to evaluate the pathological features of the seminiferous tubules and spermatogenic activity. For each patient, 10 independent seminiferous tubule cross‑sections were randomly selected for histological evaluation under light microscopy, and representative images were labeled to indicate Sertoli cells, spermatogonia, spermatocytes, and round spermatids.

### 2.3. Reverse Transcription‐Polymerase Chain Reaction (RT‐PCR)

Total RNA was extracted from clinical samples and cultured cells using the TRIzol reagent (15596026, Invitrogen, USA). RNA quantity and purity were measured by a NanoDrop spectrophotometer (ND‐2000, Thermo Fisher Scientific, USA). Complementary DNA was synthesized using a Reverse Transcription Kit (RR047A, TaKaRa, Japan). Gene expression levels of FOXP1 and GSK3B were quantified by RT‐PCR using SYBR Green Master Mix (RR820A, TaKaRa, Japan) on a StepOnePlus Real‐Time PCR System (Applied Biosystems, USA), with GAPDH as the internal control. The relative expression was calculated by the 2^−ΔΔCt^ method. The PCR primer sequences were listed as follows: FOXP1, forward primer (5′‐TGACAACAGCCAACCACAGT‐3′) and reverse primer (5′‐GGTATCAACATGCAGCGCTG‐3′); GSK3B, forward primer (5′‐GCTCCTTCCTTCCTTCCCAC‐3′) and reverse primer (5′‐CACAGACCTGCTTGCTCAGA‐3′); GAPDH, forward primer (5′‐TTCACCACCATGGAGAAGGC‐3′) and reverse primer (5′‐TGAAGTCGCAGGAGACAACC‐3′).

### 2.4. IHC Staining

Paraffin sections underwent deparaffinization, rehydration, and antigen retrieval. Endogenous peroxidase activity was blocked with 3% hydrogen peroxide. Sections were incubated overnight at 4°C with the anti‐FOXP1 antibody (ab167352, Abcam, China). Detection was performed using a biotin‐streptavidin HRP system (SP‐9000, ZSGB‐BIO, China) and visualized by the DAB substrate. Slides were counterstained with hematoxylin and imaged under a light microscope.

### 2.5. Isolation and Culture of Mouse SSCs

Primary SSCs were isolated from the testes of 6‐day‐old C57BL/6 mice. The process of primary isolation of cells was approved by the Ethics Committee of Ganzhou People’s Hospital of Jiangxi Province (Approval Number PJD2026‐014‐01). Testicular tissues were enzymatically digested with collagenase and trypsin‐EDTA, filtered, and plated in a SSC culture medium consisting of α‐MEM supplemented with 10% fetal bovine serum, GDNF, and basic fibroblast growth factor. Cells were maintained at 37°C with 5% CO_2_. SSC identity was verified by immunofluorescence staining for GFRα1 and PLZF markers (sc‐13142, Santa Cruz, USA; ab178676, Abcam, UK). Flow cytometry was performed to evaluate the purity of isolated primary mouse SSCs using SSC markers CD90, CD49f, and ID4. Freshly harvested SSCs were washed with ice‑cold PBS containing 2% FBS and aliquoted for separate detection of each marker. For surface staining, cells were incubated with CD90 (166407, Biolegend, USA) and CD49f (313611, Biolegend, USA) antibodies on ice for 30 min in the dark. For intracellular ID4 staining, cells were fixed and permeabilized, followed by incubation with the ID4 primary antibody (PA5‐26976, Invitrogen, USA) at 4°C for 30 min. At least 10,000 cellular events were collected per sample. The positive proportions of CD90^+^, CD49f^+^, and ID4^+^ cells were quantified independently. All experiments were performed in three independent biological replicates.

### 2.6. Cell Transfection With Short Hairpin RNA (shRNA)

shRNAs targeting FOXP1 (sh‐FOXP1 #1, sh‐FOXP1 #2, and sh‐FOXP1 #3) and a nontargeting shRNA (sh‐NC) were designed and synthesized by GenePharma (Shanghai, China). The sequences of sh‐FOXP1 #1, sh‐FOXP1 #2, sh‐FOXP1 #3, and sh‐NC were verified and cloned into the pLKO.1 vector (Addgene, 8453, Cambridge, MA, USA). SSCs were seeded into 6‐well plates at a density of 5 × 10^5^ cells/well and cultured until 70%–80% confluency. Transfection was performed using Lipofectamine 3000 reagent (Thermo Fisher Scientific, L3000015, Waltham, MA, USA) according to the manufacturer’s instructions. After 48 h of transfection, the cells were collected for RT‐PCR to verify the knockdown efficiency. The FOXP1 shRNA sequences were represented as follows: sh‐FOXP1 #1: 5′‐GGTTCACACTGGTAGACAA‐3′; sh‐FOXP1 #2: 5′‐CGATAGAAGCACAGCTCAA‐3′; sh‐FOXP1 #3: 5′‐GCAGCAAGTTAGTGGATTA‐3′; and sh‐NC: 5′‐TTCTCCGAACGTGTCACGT‐3′.

### 2.7. Cell Viability and Proliferation Assays

Cell viability was assessed using the Cell Counting Kit‐8 (CCK‐8, CK04, Dojindo, Japan) following the manufacturer’s instructions. Absorbance was measured at 450 nm using a microplate reader (Multiskan GO, Thermo Fisher Scientific, USA). Cell proliferation was evaluated via an EdU incorporation assay (C10310, RiboBio, China). Briefly, SSCs were seeded into 24‐well plates after transfection and cultured for 48 h. Then, 50 μM EDU was added to the culture medium, and the cells were incubated for another 2 h. The cells were fixed with 4% paraformaldehyde, permeabilized with 0.5% Triton X‐100, and stained with Apollo staining solution for 30 min in the dark. DAPI was used for nuclear staining. The stained cells were observed under a fluorescence microscope.

### 2.8. Terminal Deoxynucleotidyl Transferase dUTP Nick End Labeling (TUNEL) Assay

Apoptotic cells were detected by the TUNEL assay using the TUNEL Apoptosis Detection Kit (Roche, 11684795910, Basel, Switzerland). SSCs were seeded into 24‐well plates after transfection and cultured for 48 h. The cells were fixed with 4% paraformaldehyde, permeabilized with 0.1% Triton X‐100, and incubated with the TUNEL reaction mixture at 37°C for 1 h in the dark. DAPI was used for nuclear staining. The apoptotic rate (percentage of TUNEL‐positive cells among the total cells) was calculated under a fluorescence microscope.

### 2.9. Immunofluorescence Staining of β‐Catenin

SSCs transfected with sh‐NC or sh‐FOXP1 were fixed with 4% paraformaldehyde, permeabilized with 0.5% Triton X‐100, and blocked with 5% BSA at room temperature. Cells were incubated overnight at 4°C with a primary antibody against β‐catenin, followed by incubation with a fluorophore‐conjugated secondary antibody for 1 h at room temperature in the dark. The nuclei were counterstained with DAPI. Fluorescence images were captured using a confocal laser scanning microscope.

### 2.10. Western Blot Assay

Cells were lysed in RIPA buffer (P0013B, Beyotime, China) supplemented with protease and phosphatase inhibitors. Protein concentration was determined by the BCA assay (P0010, Beyotime, China). Equal amounts of protein (30 μg) were resolved by SDS‐PAGE and transferred onto PVDF membranes (IPVH00010, Millipore, USA). Membranes were blocked and incubated overnight at 4°C with primary antibodies against FOXP1 (ab167352, Abcam, UK), GSK3B (9832, Cell Signaling Technology, USA), PCNA (ab29, Abcam, UK), PLZF (sc‐28319, Santa Cruz, USA), ETV5 (DF13605, Affinity, China), Wnt3a (sc‐13128, Santa Cruz, USA), β‐catenin (9562, CST, USA), phosphorylated GSK3B (9323, CST, USA) and phosphorylated β‐catenin (9561, CST, USA), AXIN2 (DF6978, Affinity, China), Cyclin D1 (AF0931, Affinity, China), TCF7 (DF3168, Affinity, China), β‐actin (AF7018, Affinity, China), and GAPDH (AF7021, Affinity, China). After washing, membranes were incubated with HRP‐conjugated secondary antibodies (7074, CST, USA) and visualized using chemiluminescence reagents (P0018, Beyotime, China) with imaging on ChemiDoc XRS+ (Bio‐Rad, USA).

### 2.11. Fluorescence In Situ Hybridization (FISH) Assay

The FISH assay was performed to detect the coexpression and subcellular localization of FOXP1 and GSK3B in SSCs using the FISH Detection Kit (RiboBio, F1020, Guangzhou, China). FOXP1‐ and GSK3B‐specific probes were synthesized by RiboBio (Guangzhou, China). SSCs were seeded on coverslips, fixed with 4% paraformaldehyde, and permeabilized with 0.5% Triton X‐100. The probes were hybridized with the cells at 37°C overnight in a humidified chamber. After washing, the cells were stained with DAPI for nuclear localization. The stained cells were observed and imaged using a confocal laser scanning microscope (Leica, TCS SP8, Wetzlar, Germany).

### 2.12. Chromatin Immunoprecipitation Quantitative PCR (ChIP‐qPCR)

ChIP assays were conducted using a commercial kit (17‐371, Millipore, USA). SSCs were cross‐linked with 1% formaldehyde, quenched with glycine, and lysed. Chromatin was sheared into 200–500 bp fragments by sonication. Immunoprecipitation was performed overnight at 4°C using the anti‐FOXP1 antibody or IgG control. DNA was recovered and analyzed by qPCR with primers targeting predicted FOXP1 binding sites in the GSK3B promoter.

### 2.13. Luciferase Reporter Assay

Truncated vectors of the GSK3B promoter were constructed by cloning different fragments of the GSK3B promoter into the pGL3‐Basic vector (Promega, E1751, Madison, WI, USA). SSCs were seeded into 24‐well plates and cotransfected with the truncated promoter vectors and sh‐FOXP1 or sh‐NC using the Lipofectamine 3000 reagent. The pRL‐TK vector (Promega, E2241, Madison, WI, USA) was cotransfected as an internal reference to normalize the luciferase activity. After 48 h of transfection, the luciferase activity was detected using the Dual‐Luciferase Reporter Assay System (Promega, E1910, Madison, WI, USA) according to the manufacturer’s instructions. The relative luciferase activity was calculated as the ratio of firefly luciferase activity to Renilla luciferase activity. The full‐length human GSK3B promoter sequence containing the predicted FOXP1‐binding P3 site (−1842 to −1835 bp) was amplified and subcloned into the pGL3‐Basic luciferase vector to generate the wild‐type (WT) GSK3B promoter reporter plasmid. For site‐directed mutagenesis, the core FOXP1‐binding motif within the P3 region was mutated using a site‐directed mutagenesis kit (C214‐01, Vazyme, Nanjing, China) according to the manufacturer’s protocol. SSCs were seeded in 24‐well plates and cultured to reach 70%–80% confluence. Cells were cotransfected with either WT‐GSK3B‐promoter or MUT‐GSK3B‐promoter reporter plasmid, together with sh‐NC or sh‐FOXP1 constructs. After 48 h of transfection, cells were lysed, and firefly and Renilla luciferase activities were sequentially measured using the Dual‐Luciferase Reporter Assay System.

### 2.14. Statistical Analysis

All experiments were performed in at least three independent replicates. Data were expressed as the mean ± standard deviation (SD). Statistical significance was determined using Student’s *t*‐test or one‐way ANOVA with Tukey’s post hoc test via GraphPad Prism 8.0 (GraphPad Software, USA). A *p*‐value of <0.05 was considered statistically significant.

## 3. Results

### 3.1. FOXP1 Expression Is Significantly Downregulated in Clinical NOA Samples With Impaired Spermatogenic Morphology

HE staining was employed to characterize the pathological features of testicular tissues obtained from patients with OA and NOA (Figure 1A). In OA samples, seminiferous tubules contained spermatogenic cells at all developmental stages, with a large number of mature spermatozoa in the tubular lumen. The spermatogenic cells were arranged in a regular and orderly manner, indicating robust spermatogenic activity. In sharp contrast, NOA samples displayed extensive atrophy and necrosis of seminiferous tubules, accompanied by loose interstitial tissue with mild fibrosis, as well as necrosis and apoptosis of partial spermatocytes. To further investigate the expression pattern of FOXP1 in these clinical samples, RT‐PCR analysis was performed (Figure 1B), which revealed that FOXP1 mRNA expression was significantly downregulated in NOA samples compared with OA samples. Consistently, IHC staining further confirmed that the level of FOXP1 was markedly decreased in NOA clinical samples relative to OA counterparts (Figure 1C,D).

**Figure 1:**
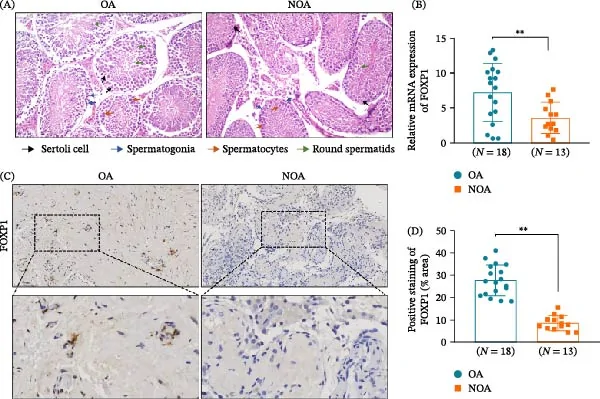
FOXP1 expression is downregulated in clinical NOA samples with impaired spermatogenesis. (A) HE staining showing pathological features of testicular tissues from OA (*N* = 18) and NOA (*N* = 13) patients. Scale bar = 100 µm. (B) RT‐PCR analysis of FOXP1 mRNA expression in OA (*N* = 18) and NOA (*N* = 13) samples. (C, D) IHC staining confirming FOXP1 protein level in OA (*N* = 18) and NOA (*N* = 13) samples. Scale bar = 100 µm. Data were presented as mean ± SD,  ^∗∗^ *p* < 0.01.

### 3.2. FOXP1 Knockdown Inhibits Viability and Proliferation of SSCs and Promotes Apoptosis

Mouse primary SSCs were isolated and identified by immunofluorescence staining using antibodies against the specific SSC markers GFRα1 and PLZF (Figure 2A). To further characterize the isolated primary mouse SSCs, the expression of the SSC markers CD90, CD49f, and ID4 was examined by flow cytometry. The percentages of CD90^+^, CD49f^+^, and ID4^+^ cells in the isolated primary cell population were greater than 90%, respectively (Figure 2B). These data confirmed the enrichment of SSCs in our primary culture. To explore the biological role of FOXP1 in SSCs, shRNAs targeting FOXP1 were constructed and transfected into SSCs, with a nontargeting shRNA (sh‐NC) serving as the negative control. RT‐PCR was used to verify the knockdown efficiency, and the results demonstrated that sh‐FOXP1 #1 and sh‐FOXP1 #2 exhibited the highest silencing efficiency among all constructed shRNAs (Figure 2C), which were selected for subsequent experimental studies. The CCK‐8 assay results showed that FOXP1 knockdown significantly suppressed the viability of SSCs compared with the sh‐NC group (Figure 2D). EDU incorporation assays further confirmed that the proliferative capacity of SSCs was remarkably inhibited after FOXP1 silencing (Figure 2E,F). In addition, TUNEL staining assays indicated that FOXP1 knockdown significantly increased the apoptotic rate of SSCs relative to the control group (Figure 2G,H). Western blot analysis was further performed to detect the expression of markers associated with SSCs proliferation and self‐renewal, and the data showed that the protein levels of PCNA, PLZF, and ETV5 were significantly reduced in FOXP1‐knockdown SSCs compared with the sh‐NC group (Figure 2I,J).

Figure 2FOXP1 knockdown inhibits SSC viability, proliferation and promotes apoptosis. (A) Immunofluorescence staining of primary SSCs using GFRα1 and PLZF antibodies (SSC‐specific markers). Scale bar = 20 µm. (B) Flow cytometry detected the expression of the SSC markers CD90, CD49f and ID4. (C) RT‐PCR verifying FOXP1 knockdown efficiency of shRNAs. (D) CCK‐8 assay detecting SSC viability after FOXP1 knockdown. (E, F) EDU incorporation assay evaluating SSC proliferation. Scale bar = 50 µm. (G, H) TUNEL staining analyzing cell apoptosis. Scale bar = 50 µm. (I, J) Western blot detecting PCNA, PLZF, and ETV5 protein levels (markers for SSC proliferation and self‐renewal). Data were presented as mean ± SD,  ^∗^ *p* < 0.05,  ^∗∗^ *p* < 0.01.

**Figure:**
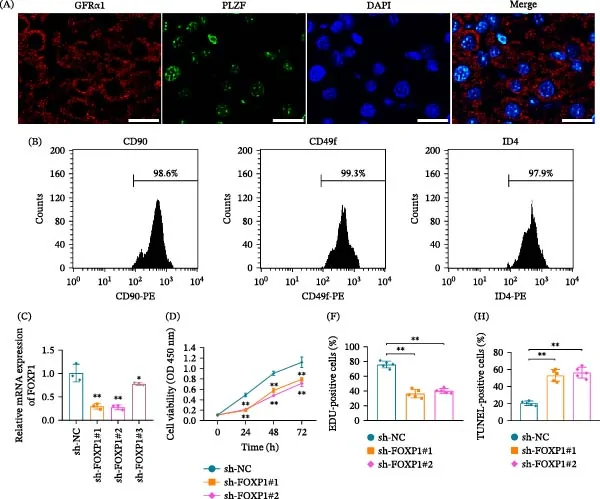

**Figure:**
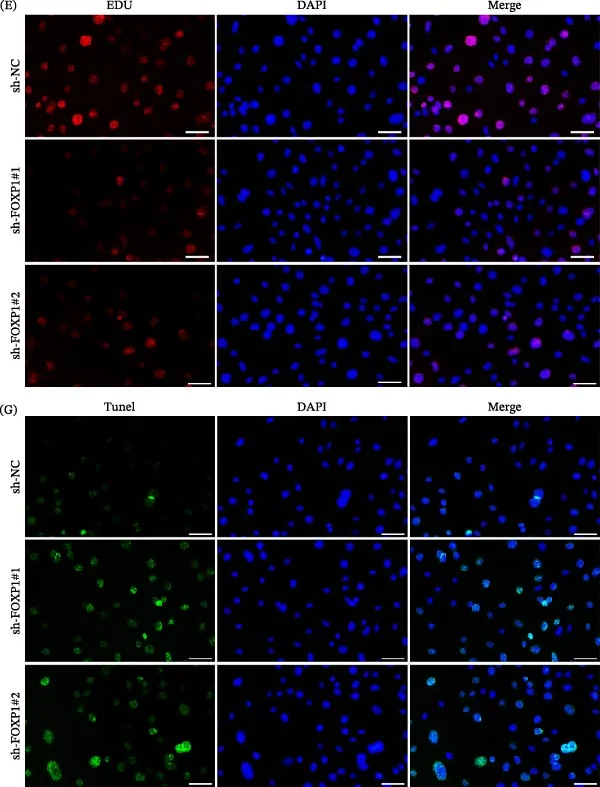

**Figure:**
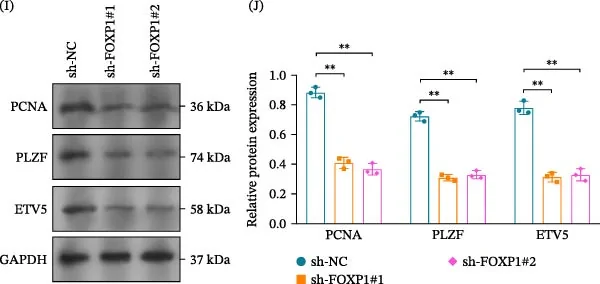

### 3.3. FOXP1 Directly Binds to the GSK3B Promoter Region and Negatively Regulates Its Transcription in SSCs

To elucidate the potential molecular mechanism underlying FOXP1‐mediated regulation of SSC proliferation, the JASPAR database (http://jaspar.genereg.net) was utilized to predict the downstream target genes of FOXP1. Bioinformatics analysis revealed that FOXP1 could potentially bind to the promoter region of GSK3B (Figure 3A). RT‐PCR detection showed that FOXP1 knockdown significantly upregulated the mRNA level of GSK3B in SSCs (Figure 3B), and this result was further validated by Western blot assays, which demonstrated a marked increase in GSK3B protein expression after FOXP1 silencing (Figure 3C,D). FISH assays confirmed the coexpression and nuclear localization of FOXP1 and GSK3B in SSCs (Figure 3E). Three potential FOXP1‐binding sites in the GSK3B promoter were predicted via the JASPAR database, and corresponding primers were designed for ChIP‐qPCR assays. Compared with the IgG control group, the GSK3B promoter sequence was significantly enriched at the P3 site (−1842 to −1835) following immunoprecipitation with an anti‐FOXP1 antibody (Figure 3F,G). To further confirm this binding relationship, truncated vectors of the GSK3B promoter were constructed, and luciferase reporter assays were performed (Figure 3H). The results showed that deletion of the −1842 nt ~0 nt region had no significant impact on the luciferase activity of the GSK3B promoter, whereas deletion of the −1835 nt ~0 nt region significantly reduced its luciferase activity. To further verify the functional requirement of the predicted FOXP1 binding site, we performed site‐directed mutagenesis targeting the core P3 binding motif (−1842 to −1835) within the GSK3B promoter. Dual‐luciferase assays were performed in SSCs transfected with WT or mutant (MUT) GSK3B promoter reporters, combined with sh‐NC or sh‐FOXP1 transfection. The result of Figure 3I indicated that in the WT promoter group, FOXP1 knockdown significantly elevated GSK3B promoter luciferase activity, consistent with FOXP1‐dependent transcriptional repression. In contrast, mutation of the P3 core binding motif completely eliminated the responsiveness of the GSK3B promoter to FOXP1 silencing, with no significant luciferase difference between sh‐NC and sh‐FOXP1 groups. These data confirm that the −1842 to −1835 region is the functional and direct binding site required for FOXP1 to repress GSK3B transcription. These findings collectively indicate that the region between −1842 and −1835 nt in the GSK3B promoter is responsible for FOXP1‐mediated transcriptional regulation of GSK3B.

**Figure 3:**
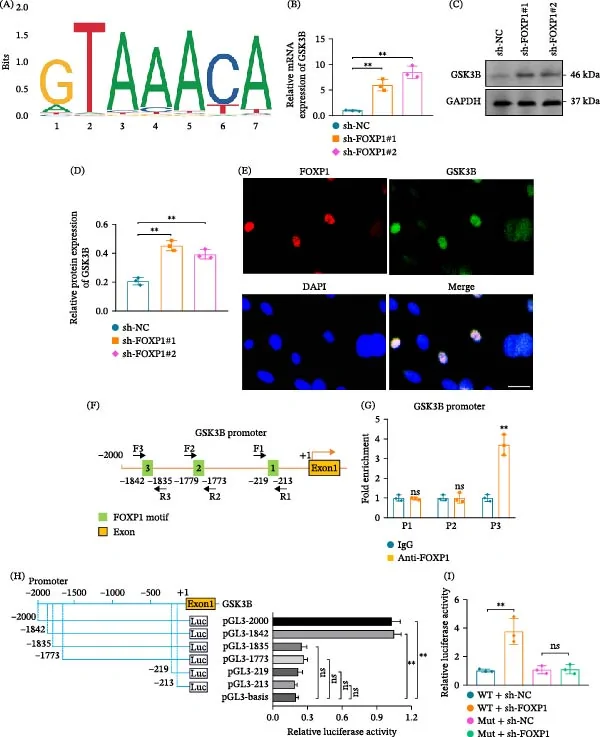
FOXP1 directly binds to GSK3B promoter and negatively regulates its transcription in SSCs. (A) JASPAR database prediction of FOXP1 binding to GSK3B promoter. (B) RT‐PCR detecting GSK3B mRNA level in FOXP1‐knockdown SSCs. (C, D) Western blot verifying GSK3B protein expression after FOXP1 silencing. (E) FISH assay showing coexpression and nuclear localization of FOXP1 and GSK3B in SSCs. Scale bar = 20 µm. (F, G) ChIP‐qPCR assay confirming FOXP1 binding to GSK3B promoter at P3 site (−1842 to −1835). (H) Luciferase reporter assay identifying the FOXP1‐binding region in GSK3B promoter. (I) Dual‐luciferase assay in SSCs transfected with WT or P3‐mutated GSK3B promoter reporters plus sh‐NC or sh‐FOXP1. Relative luciferase activity (firefly/Renilla) was measured. Data were presented as mean ± SD, ns = nonsignificant,  ^∗∗^ *p* < 0.01.

### 3.4. FOXP1 Knockdown Suppresses Activation of the Wnt/β‐Catenin Signaling Pathway in SSCs

To evaluate the effect of FOXP1 silencing on the Wnt/β‐catenin signaling pathway, Western blot assays were performed to detect the expression levels of Wnt3a, GSK‐3β, phosphorylated GSK‐3β (p‐GSK‐3β), β‐catenin, and phosphorylated β‐catenin (p‐β‐catenin) in SSCs transfected with sh‐FOXP1 #1, sh‐FOXP1 #2, or sh‐NC (Figure 4A–F). Compared with the sh‐NC group, FOXP1 knockdown significantly decreased the protein expression of Wnt3a and β‐catenin while remarkably increasing the expression levels of GSK‐3β, p‐GSK‐3β, and p‐β‐catenin. In addition, to comprehensively verify the regulatory effect of FOXP1 on canonical Wnt/β‐catenin signaling, we examined key downstream effectors and β‐catenin subcellular localization in FOXP1‐knockdown SSCs. Western blot results (Figure 4G–J) showed that the protein levels of Wnt target genes, including AXIN2, Cyclin D1, and TCF7, were significantly decreased in the sh‐FOXP1 group compared with the sh‐NC control. Consistent with the reduced Wnt target expression, immunofluorescence staining (Figure 4K) revealed prominent nuclear accumulation of β‐catenin in control SSCs, whereas FOXP1 knockdown markedly diminished nuclear β‐catenin fluorescence intensity. These results suggest that FOXP1 silencing significantly inhibits the activation of the Wnt/β‐catenin signaling pathway in SSCs.

Figure 4FOXP1 knockdown suppresses Wnt/β‐Catenin signaling pathway activation in SSCs. (A–F) Western blot detecting protein levels of Wnt3a, GSK‐3β, p‐GSK‐3β, β‐catenin and p‐β‐catenin in SSCs transfected with sh‐FOXP1 #1, sh‐FOXP1 #2 or sh‐NC. (G–J) Western blot and quantitative analysis detected the protein levels of canonical Wnt downstream markers AXIN2, Cyclin D1, and TCF7 in sh‐NC and sh‐FOXP1 SSCs. (K) Immunofluorescence staining of β‐catenin. Scale bar = 20 µm. Data were presented as mean ± SD, ns = nonsignificant,  ^∗∗^ *p* < 0.01.

**Figure:**
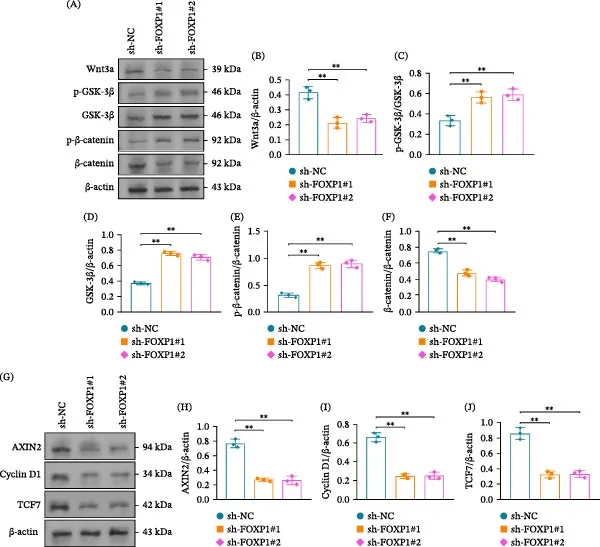

**Figure:**
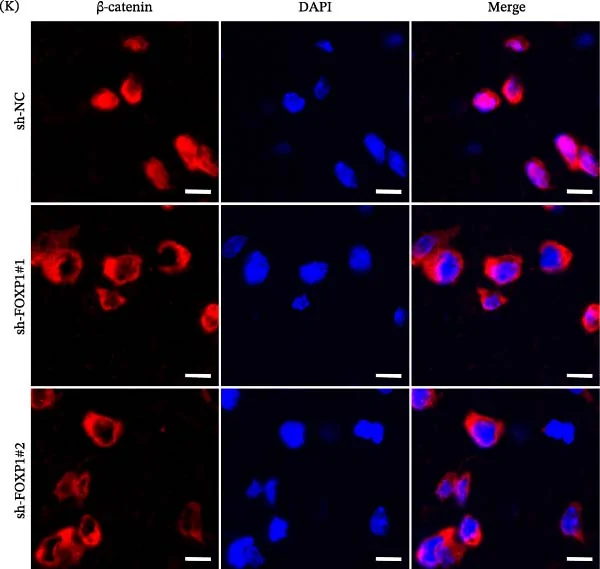

### 3.5. Activation of the Wnt/β‐Catenin Pathway Reverses the Biological Effects of FOXP1 Knockdown on SSCs

To further confirm whether the biological functions of FOXP1 in SSCs are mediated by the Wnt/β‐catenin pathway, FOXP1‐knockdown SSCs were treated with LiCl, a specific activator of the Wnt/β‐catenin signaling pathway, and changes in cell viability, proliferation, and apoptosis were subsequently detected. Consistent with the aforementioned results, FOXP1 knockdown significantly inhibited SSC viability (Figure 5A) and proliferation (Figure 5B,C) while promoting cell apoptosis (Figure 5D,E) compared with the sh‐NC group. Notably, LiCl treatment significantly attenuated the inhibitory effects of FOXP1 knockdown on SSC viability and proliferation and also reversed the pro‐apoptotic effect of FOXP1 silencing in SSCs. Furthermore, knockdown of FOXP1 inhibited the expression of PCNA, PLZF, and ETV5 (Figure 5F–I). While LiCl treatment significantly reversed the effect of sh‐FOXP1 and significantly promoted the expression of PCNA, PLZF, and ETV5. To further validate the specific regulatory role of FOXP1 in SSC proliferation and confirm its dependence on β‐catenin signaling, we performed FOXP1 overexpression (FOXP1‐OE) combined with β‐catenin knockdown rescue experiments. The EDU proliferation assay (Figure 5J,K) showed that FOXP1‐OE significantly increased the EDU‐positive ratio of SSCs compared with the Vector control group, indicating enhanced cell proliferation. In contrast, β‐catenin knockdown largely abolished the pro‐proliferative effect induced by FOXP1‐OE. Consistently, Western blot results (Figure 5L–O) demonstrated that FOXP1‐OE markedly upregulated the expression of the level of PCNA, PLZF, and ETV5. However, knockdown of β‐catenin effectively reversed the upregulation of these markers induced by FOXP1‐OE. These data indicate that the Wnt/β‐catenin signaling pathway is involved in FOXP1‐mediated regulation of SSC biological behaviors.

Figure 5Activation of Wnt/β‐Catenin pathway reverses the effects of FOXP1 knockdown on SSCs. (A) CCK‐8 assay detecting SSC viability. (B, C) EDU incorporation assay evaluating SSC proliferation. Scale bar = 50 µm. (D, E) TUNEL staining analyzing SSC apoptotic rate. Scale bar = 50 µm. (F–I) Western blot detecting PCNA, PLZF, and ETV5 protein levels. (J, K) EDU incorporation assay evaluating SSC proliferation. Scale bar = 50 µm. (L–O) Western blot detecting PCNA, PLZF, and ETV5 protein levels. Data were presented as mean ± SD,  ^∗∗^ *p* < 0.01.

**Figure:**
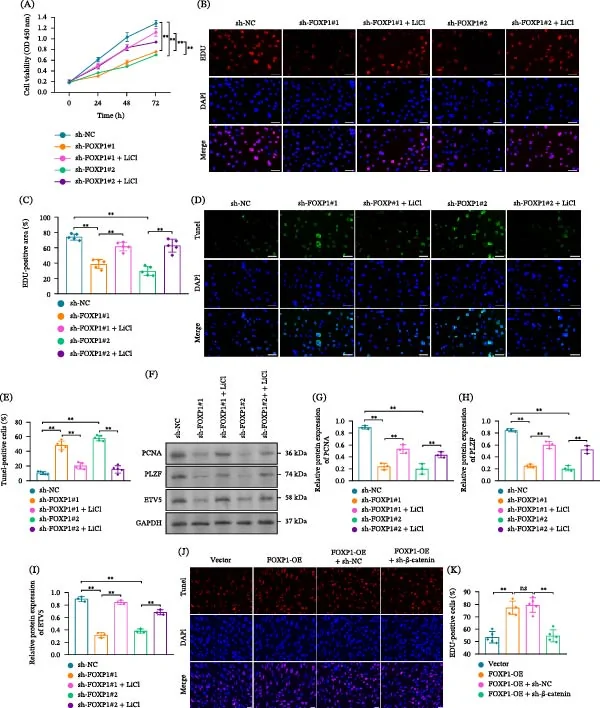

**Figure:**
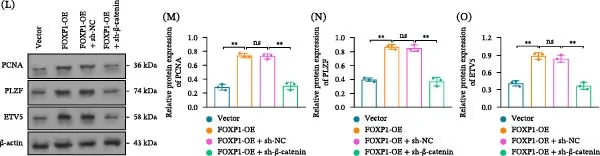

## 4. Discussion

NOA, characterized by impaired spermatogenesis and the absence of sperm in ejaculate, is a major cause of male infertility with complex pathogenic mechanisms that remain not fully elucidated [15, 16]. Emerging evidence indicates that dysregulated expression of transcription factors plays a pivotal role in the progression of NOA as these factors are essential for maintaining the normal development and function of SSCs, the foundation of spermatogenesis [17].

FOXP1, a member of the forkhead box (FOX) transcription factor family, has been widely reported to regulate cell proliferation, apoptosis, and differentiation in various tissues and cell types [18, 19]. Our observation demonstrated that FOXP1 mRNA and protein levels are significantly decreased in NOA samples compared with OA controls. Notably, FOXP1 knockdown has been shown to inhibit the viability and proliferation of various stem cells [20, 21], which supports our results that silencing FOXP1 impairs SSC viability and proliferation while promoting apoptosis. This suggests that FOXP1 may serve as a key regulator of the SSC function, and its downregulation in NOA may contribute to the impairment of spermatogenic activity.

To further explore the molecular mechanism underlying FOXP1‐mediated regulation of SSCs, we identified GSK3B as a direct downstream target of FOXP1. GSK3B, a serine/threonine kinase, is a well‐known component of the Wnt/β‐catenin signaling pathway [22]. Our finding revealed that FOXP1 knockdown upregulates GSK3B expression and impairs SSCs function. ChIP‐qPCR and luciferase reporter assays confirmed that FOXP1 directly binds to the GSK3B promoter to negatively regulate its transcription. The Wnt/β‐catenin signaling pathway is closely associated with spermatogenesis, and its dysregulation has been implicated in NOA pathogenesis [23]. Our results showed that FOXP1 knockdown inhibits the activation of the Wnt/β‐catenin pathway, which is supported by previous studies demonstrating that GSK3B overexpression suppresses Wnt/β‐catenin signaling by promoting β‐catenin phosphorylation and degradation [24]. Furthermore, activation of the Wnt/β‐catenin pathway with LiCl reversed the inhibitory effects of FOXP1 knockdown on SSCs, indicating that FOXP1 regulates SSC function through the GSK3B‐mediated Wnt/β‐catenin signaling pathway. This finding links FOXP1 to the well‐established Wnt/β‐catenin pathway in spermatogenesis, providing new insights into the molecular mechanisms of NOA. Importantly, the potential translational implication of targeting the Wnt/β‐catenin pathway for NOA treatment require cautious interpretation due to inherent safety risks. Constitutive and aberrant hyperactivation of canonical Wnt/β‐catenin signaling is a well‐documented oncogenic driver that promotes tumorigenesis in multiple human malignancies [25]. Accumulating clinical evidence also indicates that patients with NOA possess a significantly elevated long‐term risk of testicular neoplasms, creating a critical safety concern for Wnt‐based therapeutic intervention. In this study, we demonstrated that restoration of Wnt/β‐catenin signaling could rescue the defective proliferation and survival of FOXP1‐deficient SSCs at the cellular level. However, this finding does not support the feasibility of global, persistent Wnt activation for NOA clinical treatment. Nonspecific systemic activation of the Wnt pathway may disrupt tissue homeostasis and potentiate malignant transformation in testicular germ cells. Therefore, we emphasize that future translational applications should prioritize transient, spermatogenic cell‐specific modulation of the FOXP1‐GSK3B‐Wnt/β‐catenin axis rather than broad and permanent pathway activation. Rigorous preclinical safety evaluation and targeted delivery strategy optimization are indispensable before considering any clinical therapeutic transformation of this regulatory axis.

Despite these findings, this study has certain limitations. First, the sample size of clinical NOA samples is relatively small, and further studies with larger cohorts are needed to validate the downregulation of FOXP1 in NOA. Second, the in vivo role of FOXP1 in spermatogenesis was not investigated, which should be addressed in future animal experiments. Notably, the current study has inherent limitations arising from the biological heterogeneity of NOA. NOA is a pathologically heterogeneous disorder mainly categorized into SCO syndrome, MA, and HS, each exhibiting distinct spermatogenic impairment patterns and molecular regulatory characteristics [26]. In this study, all enrolled NOA samples were integrated into a single group for comparative analysis with OA samples. Given the limited number of NOA clinical samples (*n* = 13), subgroup analysis based on different histopathological subtypes would result in extremely small sample sizes for each stratified group, leading to insufficient statistical power to obtain stable and credible subgroup differences. Therefore, we adopted a pooled grouping strategy for the primary analysis focused on exploring the overall differential expression and functional role of FOXP1 in NOA‐associated spermatogenic dysfunction. Nevertheless, we cannot rule out the possibility that the aberrant expression of FOXP1 and its regulatory effects on SSC biology may vary across different NOA pathological subtypes. Future studies with expanded clinical sample sizes are required to conduct stratified analyses of different NOA subtypes, further clarifying the subtype‐specific regulatory characteristics of the FOXP1‐GSK3B‐Wnt/β‐Catenin axis and improving the specificity and comprehensiveness of the research conclusions. Another important clinical limitation of this study originates from the multifocal histological heterogeneity of NOA testicular tissues and the inherent constraints of testicular biopsy sampling. In the present work, all human testicular samples were collected from the unilateral testis, and only two spatially separated biopsy fragments were acquired per patient for histological staining and molecular detection, respectively. It is well‐established that NOA testes often display regional discrepancies in spermatogenic status, with normal, hypoplastic, or completely degenerated seminiferous tubules coexisting in different areas of the same testis [27]. Accordingly, the FOXP1 expression levels detected in the sampled biopsy regions may not fully reflect the overall spermatogenic status and molecular characteristics of the entire testicular tissue. Such sampling bias is unavoidable in most human testicular biopsy‐based infertility studies. Future investigations combining multisite biopsy sampling or whole‐tissue section analysis will help verify the global expression pattern of FOXP1 in NOA testes and further validate its clinical relevance. Furthermore, an unavoidable limitation of this study lies in the species difference between clinical human samples and mechanistic cellular experiments. Although our clinical correlation analysis was performed using human testicular tissues, all functional verification and molecular mechanism explorations were conducted in primary mouse SSCs. The core Wnt/β‐Catenin signaling cascade and the regulatory function of FOXP family transcription factors are broadly conserved in mammalian spermatogenesis, supporting the biological rationality of our mouse‐based mechanistic model. However, there are substantial interspecies differences between mouse and human spermatogonia including divergent promoter binding preferences, distinct transcriptional regulatory networks, and varied responses to upstream signaling modulation. Therefore, the direct causal mechanism established in mouse SSCs cannot be completely and indiscriminately extrapolated to human SSCs. Future functional validation in human germ cell models, such as human SSC lines or testicular organoids, is required to further confirm the conservation of the FOXP1‐GSK3B‐Wnt/β‐Catenin regulatory axis in human spermatogenesis and consolidate the translational significance of our findings in human NOA pathogenesis. In addition, although we focused on the GSK3B–Wnt axis, FOXP1 can regulate additional targets (e.g., cell‐cycle genes, Notch, or TGF‐β components) that may also influence the SSC fate. Comprehensive transcriptomic profiling (RNA‐seq) of FOXP1‐manipulated SSCs will be needed to uncover the broader regulatory network. In conclusion, our findings demonstrate that the FOXP1‐GSK3B‐Wnt/β‐catenin signaling axis plays an essential role in maintaining SSC homeostasis and spermatogenic function. Dysregulation of this axis contributes to impaired spermatogenesis in NOA patients. This study provides novel mechanistic insights into the molecular pathogenesis of NOA, while further clinical validation and preclinical functional verification are required to determine its potential translational value.

## Author Contributions

Conception and design of the research, revision of manuscript for important intellectual content: Jin‐Hua Wu and Hui‐Liang Zhou. Acquisition of data: Jin‐Hua Wu, Qing Feng and Feng Lin. Analysis and interpretation of data: Jiang‐Ping Xie and Pei‐Yan Liu. Statistical analysis: Yan‐Hua Wang. Drafting the manuscript: Jin‐Hua Wu, Qing Feng, and Feng Lin.

## Funding

The authors declare that this research did not receive any funding.

## Ethics Statement

The collection of clinical samples was approved by the Ethics Committee of Ganzhou People’s Hospital of Jiangxi Province (Approval Number PJB2025‐279‐01). The process of primary isolation of SSCs was approved by the Ethics Committee of Ganzhou People’s Hospital of Jiangxi Province (Approval Number PJD2026‐014‐01).

## Conflicts of Interest

The authors declare no conflicts of interest.

## Supporting Information

Additional supporting information can be found online in the Supporting Information section.

## Supporting information


**Supporting Information** Table S1: Pathological classification of NOA and OA patients.

## Data Availability

The data utilized and/or analyzed throughout this research are available from the corresponding author upon reasonable request.
