# Supplementary material for: FOXP1 Promotes Spermatogonial Stem Cell Proliferation by Transcriptionally Repressing GSK3B to Activate the Wnt/β‐Catenin Signaling Pathway
Source: Stem Cells Int. 2026 Oct 19;2026:1109631. doi: 10.1155/sci/1109631 (PMC13681013; doi:10.1155/sci/1109631)
Supplement: Supplementary file 1 [file SCI-2026-1109631-s001.docx]

| **Patient ID** | **Group** | **Age (years)** | **Histopathological subtype** |
| --- | --- | --- | --- |
| OA-01 | OA | 35 | normal spermatogenesis |
| OA-02 | OA | 28 | normal spermatogenesis |
| OA-03 | OA | 30 | normal spermatogenesis |
| OA-04 | OA | 34 | normal spermatogenesis |
| OA-05 | OA | 39 | normal spermatogenesis |
| OA-06 | OA | 36 | normal spermatogenesis |
| OA-07 | OA | 33 | normal spermatogenesis |
| OA-08 | OA | 37 | normal spermatogenesis |
| OA-09 | OA | 42 | normal spermatogenesis |
| OA-10 | OA | 29 | normal spermatogenesis |
| OA-11 | OA | 41 | normal spermatogenesis |
| OA-12 | OA | 31 | normal spermatogenesis |
| OA-13 | OA | 29 | normal spermatogenesis |
| OA-14 | OA | 33 | normal spermatogenesis |
| OA-15 | OA | 31 | normal spermatogenesis |
| OA-16 | OA | 41 | normal spermatogenesis |
| OA-17 | OA | 29 | normal spermatogenesis |
| OA-18 | OA | 32 | normal spermatogenesis |
| NOA-01 | NOA | 35 | MA |
| NOA-02 | NOA | 39 | HS |
| NOA-03 | NOA | 30 | HS |
| NOA-04 | NOA | 33 | SCO |
| NOA-05 | NOA | 38 | MA |
| NOA-06 | NOA | 42 | SCO |
| NOA-07 | NOA | 40 | SCO |
| NOA-08 | NOA | 32 | MA |
| NOA-09 | NOA | 36 | MA |
| NOA-10 | NOA | 41 | HS |
| NOA-11 | NOA | 27 | SCO |
| NOA-12 | NOA | 36 | MA |
| NOA-13 | NOA | 33 | SCO |

**Table. S1** Pathological classification of NOA and OA patients

MA: maturation arrest; HS: hypospermatogenesis; SCO: Sertoli‑cell‑only syndrome.
